# Awareness and Use of the After-Visit Summary Through a Patient Portal: Evaluation of Patient Characteristics and an Application of the Theory of Planned Behavior

**DOI:** 10.2196/jmir.5207

**Published:** 2016-04-13

**Authors:** Srinivas Emani, Michael Healey, David Y Ting, Stuart R Lipsitz, Harley Ramelson, Vladimir Suric, David W Bates

**Affiliations:** ^1^ Division of General Internal Medicine, Department of Medicine Brigham and Women's Hospital Harvard Medical School Boston, MA United States; ^2^ Brigham and Women's Physicians Organization Brigham and Women's Hospital Boston, MA United States; ^3^ Massachusetts General Physicians Organization Massachusetts General Hospital Boston, MA United States; ^4^ Information Services Partners HealthCare Boston, MA United States; ^5^ Department of Healthcare Policy and Management Harvard School of Public Health Boston, MA United States

**Keywords:** patient portal, after-visit summary (AVS), meaningful use, electronic health records (EHRs), beliefs

## Abstract

**Background:**

Patient portals are being used to provide a clinical summary of the office visit or the after-visit summary (AVS) to patients. There has been relatively little research on the characteristics of patients who access the AVS through a patient portal and their beliefs about the AVS.

**Objective:**

The aim was to (1) assess the characteristics of patients who are aware of and access the AVS through a patient portal and (2) apply the Theory of Planned Behavior (TPB) to predict behavioral intention of patients toward accessing the AVS provided through a patient portal.

**Methods:**

We developed a survey capturing the components of TPB (beliefs, attitude, perceived norm, and perceived behavioral control). Over a 6-month period, patients with a patient portal account with an office visit in the previous week were identified using our organization’s scheduling system. These patients were sent an email about the study and a link to the survey via their portal account. We applied univariate statistical analysis (Pearson chi-square and 1-way ANOVA) to assess differences among groups (aware/unaware of AVS and accessed/did not access AVS). We reported means and standard deviations to depict belief strengths and presented correlations between beliefs and attitude, perceived norm, and perceived behavioral control. We used hierarchical regression analysis to predict behavioral intention toward accessing the AVS through the patient portal.

**Results:**

Of the 23,336 patients who were sent the survey, 5370 responded for a response rate of 23.01%. Overall, 76.52% (4109/5370) were aware that the AVS was available through the patient portal and 54.71% of those (2248/4109) accessed the AVS within 5 days of the office visit. Patients who accessed the AVS had a greater number of sessions with the portal (mean 119, SD 221.5) than those who did not access the AVS (mean 79.1, SD 123.3, *P*<.001); the difference was not significant for awareness of the AVS. The strongest behavioral beliefs with accessing the AVS were being able to track visits and tests (mean 2.53, SD 1.00) followed by having medical information more readily accessible (mean 2.48, SD 1.07). In all, 56.7% of the variance in intention to access the AVS through the portal was accounted for by attitude, perceived norm, and perceived behavioral control.

**Conclusions:**

Most users of a patient portal were aware that the AVS was accessible through the portal. Patients had stronger beliefs about accessing the AVS with the goal of timely and efficient access of information than with engaging in their health care. Interventions to improve patient access of the AVS can focus on providers promoting patient beliefs about the value of the AVS for tracking tests and visits, and timely and efficient access of information.

## Introduction

The adoption and use of patient portals tethered to electronic health records (EHRs) has accelerated in the last decade. A primary driver of this growth has been the Medicare and Medicaid EHR Incentive Program, widely referred to as the EHR Meaningful Use (MU) program, introduced in the Health Information Technology for Economic and Clinical Health (HITECH) provision of the American Recovery and Reinvestment Act of 2009 [1,2]. The objectives of the MU program are to increase the adoption of EHRs and the meaningful use of EHRs to improve delivery of care, decrease medical errors, improve efficiency of care, and enhance patient centeredness of care [2]. The MU program is being implemented in three stages with the criteria for achieving meaningful use of the EHR becoming more rigorous with each stage. Patient portals are expected to play a key role in the MU program by providing patients with timely and efficient access to information, engaging patients in their care, and enhancing patient centeredness of care [3,4]. One of the core objectives of the MU program is to allow patients to view online and download their health information, such as test results, problem and medication lists, and medication allergies. For example, the Blue Button initiative has been implemented by a number of organizations to allow patients to download a copy of their health information by clicking on a blue circle on the patient portal page [5].

Patient portals are also being used to provide a clinical summary of the office visit or the after-visit summary (AVS) to patients. The Centers for Medicare and Medicaid Services (CMS) has defined the AVS as a clinical summary that “provides a patient with relevant and actionable information and instructions” such as the provider’s office contact information, date and location of visit, an updated medication list, updated vitals, reason(s) for visit, procedures and other instructions based on clinical discussions that took place during the office visit, any updates to a problem list, immunizations or medications administered during visit, summary of topics covered/considered during visit, and time and location of next appointment/testing, if scheduled [6]. Stage 1 of the MU program specified that the AVS should be provided to patients for more than 50% of all office visits within three business days.

The AVS requirement was controversial in the physician community and, in spite of the Stage 1 requirement of the MU program for the provision of the AVS, there has been relatively little research on how patients view the AVS. In a survey of the printed version of the AVS provided at an office visit, Neuberger and colleagues [7] reported that 88% of respondents said the information on the AVS was easy to understand and 84% said that the AVS was helpful. Chung and colleagues [8] reported similar results in their survey of a printed version of the AVS: 93% of patients agreed that they understood the information on the AVS and 93% agreed that having the AVS was helpful. Ralston and colleagues [9] reported that the AVS was the fastest growing use of their organization’s patient portal and may reflect the patient’s desire for information about their care plan and needs. Pavlik and colleagues [10] found patient satisfaction with the MU version of the AVS did not differ significantly from other content versions of the AVS. In this study, 30% of the patients reported that they plan to keep the AVS for their next appointment.

Although these studies provide some understanding of patient opinions about the AVS, we know relatively little about predictors of patient access to the AVS through the patient portal, such as do younger and more highly educated patients access (ie, retrieve) the AVS more through the patient portal compared to older and less educated patients, and what role does patient experience with the patient portal play in their accessing the AVS through the portal? There is also a lack of evidence on patient beliefs and attitude toward accessing the AVS. Do patients believe that accessing the AVS through the patient portal will provide information in a timely manner, allow them to track their visits and tests, and reinforce their provider’s instructions? This study contributes to the sparse literature on patient portals and the AVS by addressing the following objectives: (1) assess the characteristics of patients who are aware of and access (retrieve) the AVS through a patient portal and (2) apply the Theory of Planned Behavior (TPB) to evaluate beliefs, attitude, perceived norm, perceived behavioral control, and predict behavioral intention of patients toward accessing the AVS provided through a patient portal.

## Methods

### Theoretical Model

In a previous study, we pointed to the lack of application of theoretical models in the study of patient adoption and use of patient portals [11]. In that study, Rogers’ Diffusion of Innovation model was successfully applied to assess patient perceptions of a patient portal. The perceived attributes of ease of use and relative advantage of the portal emerged as significant predictors of portal adoption and value. Another technology adoption model, the Unified Theory of Adoption and Use of Technology, was successfully applied by Turvey and colleagues [12] in their study of use of the Blue Button at the Department of Veterans Affairs patient portal, MyHealtheVet. In that study, factors such as knowledge and usability of the Blue Button emerged as significant barriers to the use of the Blue Button associated with the patient portal. However, we continued to find a lack of application of theoretical models in the study of patient portals. Furthermore, there was a need for theoretical models that could predict behavioral intention and behavior toward the use of patient portals and the specific functionality associated with portals such as the AVS. The application of such models could also yield useful prescriptive implications for practitioners who are interested in improving patient use of patient portals.

In this study, we applied a prominent theoretical model, the Theory of Planned Behavior (TPB), to patient portals with a focus on predicting patients’ behavioral intention toward accessing the AVS provided through the portal. According to TPB, three major factors lead to the formation of a patient’s intention to perform a behavior: attitude toward the behavior, perceived norm, and perceived behavioral control [13]. Underlying each of these factors is a set of beliefs: behavioral beliefs about the positive or negative consequences of accessing the AVS (determines attitude), normative beliefs that important people would approve or disapprove of the patient accessing the AVS or that these referents themselves are accessing the AVS (determines perceived norm), and control beliefs that facilitate or impede the patient accessing the AVS through the patient portal (determines perceived behavioral control). Behavioral intention is a direct predictor of the behavior of accessing the AVS through the patient portal. However, factors such as skills and environmental factors may influence the relationship between intention and behavior. For example, a patient may lack the computer skills to access the AVS through the portal even if they form an intention to access the AVS. Finally, background factors such as education, age, and race may indirectly influence beliefs about accessing the AVS. Over the last three decades, TPB has been successfully applied to predict intention across a wide range of health and risk behaviors, including exercise, breast self-examination, eating a low-fat diet, condom use, alcohol consumption, smoking, and using drugs [13,14].

### Survey Instrument

To apply TPB to predict patients’ intention to access the AVS through the patient portal, we developed and implemented a cross-sectional survey that captured the different components of TPB. We followed the approach recommended by Fishbein and Ajzen [13] in developing our survey instrument. First, we conducted a pilot study to gather data on the beliefs related to patients’ accessing the AVS through the patient portal. In TPB, the behavior of interest is defined by four elements through the principle of compatibility: the action performed, the target at which the action is directed, the context in which it is performed, and the time at which it is performed [13]. In our pilot study, we defined the behavior of interest as accessing (action) the AVS (target) through the patient portal (context) within 7 days of the visit (time). Our pilot survey consisted of three questions related to this behavior of interest: (1) asking patients to list the advantages and disadvantages of accessing the AVS through the patient portal within 7 days of the visit to identify behavioral beliefs, (2) asking patients to list individuals or groups who would approve or disapprove of their accessing the AVS through the patient portal to identify injunctive normative beliefs, and (3) asking patients to list the factors or circumstances that would make it easy or difficult for them to access the AVS through the patient portal to identify control beliefs. Based on the responses to the pilot survey, we created the items for our survey. Additionally, we changed the time component for accessing the AVS to within 5 days of the visit. Multimedia Appendix 1 lists the survey items on TPB we developed for our study.

### Recruitment

The study was implemented in the ambulatory care practices of an academic medical center affiliated with Partners HealthCare, an integrated delivery system located in Eastern Massachusetts. Partners developed its own patient portal, Patient Gateway, following its strategy of developing and implementing its own EHR, the Longitudinal Medical Record. The patient portal has functionality similar to other vendor portals, including requests for appointments, prescription refills and referrals, access to certain components of the EHR (eg, laboratory results), and secure messaging with the practice and provider. The AVS is made available to patients through the portal. Patient portal transactions are stored permanently in the Partners clinical information systems and can be accessed for research purposes after institutional review board approval. Over a 6-month period, patients with a patient portal account and an office visit in the previous week were identified using the Partners scheduling system. These patients were sent an email about the study through their portal account. The email included a link to the survey. After 7 days, patients were sent a reminder email with another link to the survey. Patients were not compensated for the survey. All study materials and methods were approved by the Partners Health Care Institutional Review Board.

### Statistical Analysis

We present frequencies and means of sociodemographic characteristics and factors related to portal experience for the different groups: aware of AVS / unaware of AVS and accessed AVS / did not access AVS. To assess for differences between the groups, we conducted chi-square tests for categorical data (Pearson chi-square for dichotomous and nominal variables) and robust 1-way ANOVA for continuous variables. We employed multiple regression analysis using a forced entry method to assess predictors of patient satisfaction with the AVS. To test the application of TPB, we computed Cronbach alpha for the major factors (attitude, perceived norm, and perceived behavioral control). We then created scales for each factor using the mean of the scores of the items for each scale. We also computed Cronbach alpha and created a scale for behavioral intention to access the AVS through the patient portal. We present means and standard deviations for the belief items captured through our survey and correlations of the belief items with respective factors. Finally, we conducted hierarchical regression analysis to predict behavioral intention from the major factors of TPB as well as external factors such as sociodemographics and portal experience.

## Results

### Response Rate

Of the 23,336 patients who received the online survey, 5370 responded for a response rate of 23.01%. Table 1 shows the characteristics of the responders and nonresponders. Overall, 61.79% (3318/5370) of responders and 62.51% (11,231/17,966) of nonresponders were female; 90.76% (4874/5370) of responders were white compared to 86.44% (15,530/17,966) of nonresponders (*P*<.001). Responders were older (mean 56.6, SD 14.0 years) than nonresponders (mean 50.4, SD 15.4 years, *P*<.001). Responders also had a portal account for a longer time (mean 3.5, SD 3.3 years) compared to nonresponders (mean 2.8, SD 2.9 years) and sent a greater number of messages (mean 7.7, SD 20.1) compared to nonresponders (mean 4.3, SD 13.8). Given the large sample sizes of responders and nonresponders, the statistically significant differences should be viewed with caution.

**Table 1:** Characteristics of responders and nonresponders (N=23,336).

Characteristics	Responders n=5370	Nonresponders n=17,966	*P*
Gender (female), n (%)	3318 (61.79)	11,231 (62.51)	.34
Age (years), mean (SD)	56.6 (14.0)	50.4 (15.4)	<.001
Race (white), n (%)	4874 (90.76)	15,530 (86.44)	<.001
Selected problems on problem list,^a^ mean (SD)	2.1 (1.2)	2.0 (1.1)	<.001
Years with patient portal account, mean (SD)	3.5 (3.3)	2.8 (2.9)	<.001
Sessions with patient portal, mean (SD)	100.4 (173.5)	57.9 (109.6)	<.001
Messages sent via patient portal, mean (SD)	7.7 (20.1)	4.3 (13.8)	<.001

^a^ The selected problems included hypertension, hyperlipidemia, diabetes, cancer (any), coronary artery disease, congestive heart failure, asthma, osteoarthritis, rheumatoid arthritis, and depression.

### Awareness and Access of the After-Visit Summary

Among the 5370 responders of the survey, 4109 (76.52%) reported that they were aware of the availability of the AVS through the patient portal and 1169 (21.77%) reported that they were not aware of the availability of the AVS through the portal (92/5370, 1.71% did not respond to this question). Table 2 shows characteristics of patients who were aware and unaware of the AVS; 61.35% (2521/4109) of female patients were aware of the AVS and 63.99% (748/1169) of female patients were unaware of the AVS. Patients who were aware of the AVS had a mean age of 56.4 (SD 14.0) years compared to a mean age of 57.1 (SD 13.7) years for patients who were unaware of the AVS. In all, 90.58% (3722/4109) and 91.79% (1073/1169) of patients were white among patients who were aware and unaware of the AVS, respectively. In all, 67.78% (2452/3618) of patients who were aware of the AVA and 67.7% (674/995) who were unaware of the AVS reported their marital status as married or an unmarried couple; 51.11% (1873/3655) of patients who were aware of the AVS and 50.65% (508/1003) who were unaware of the AVS reported their health status as very good or excellent. The proportion of patients who reported that they were a 4-year college graduate or more was lower in the aware group (67.54%, 2461/3644) compared to the unaware group (79.42%, 795/1001, *P*<.001). The proportion of patients who reported total household income from all sources as US $75,000 or more was also lower in the aware group (62.55%, 2036/3255) compared to the unaware group (66.4%, 570/858, *P*=.04). Patients who were aware of the AVS had a portal account for a mean of 3.4 (SD 3.3) years compared to a mean of 4.0 (SD 3.4) years for patients who were not aware of the AVS (*P*<.001). The two groups did not differ on the number of sessions with the portal or the number of messages sent via the portal. Satisfaction with the portal was significantly higher in the AVS aware group (45.28%, 164/3653 reporting excellent) compared to the unaware group (30.1%, 299/993, *P*<.001).

**Table 2:** Characteristics of respondents who were aware/unaware of the after-visit summary (AVS).

Characteristics	Aware of AVS n=4109	Unaware of AVS n=1169	*P*
Gender (female), n (%)	2521 (61.35)	748 (63.99)	.10
Age (years), mean (SD)	56.4 (14.0)	57.1 (13.7)	.16
Race (white), n (%)	3722 (90.58)	1073 (91.79)	.21
Education (≥4-year college degree), n (%)	2461/3644 (67.54)	795/1001 (79.42)	.001
Income (≥US$75,000),^a^ n (%)	2036/3255 (62.55)	570/858 (66.4)	.04
Marital status (married or unmarried couple), n (%)	2452/3618 (67.78)	674/995 (67.7)	.98
Health status (very good or excellent),^b^ n (%)	1873/3665 (51.11)	508/1003 (50.65)	.80
Selected problems on problem list,^c^ mean (SD)	2.1 (1.2)	2.2 (1.2)	.36
Years with patient portal account, mean (SD)	3.4 (3.3)	4.0 (3.4)	.001
Sessions with patient portal, mean (SD)	101.8 (185.4)	97.4 (128.6)	.44
Messages sent via patient portal, mean (SD)	7.5 (19.7)	8.5 (20.9)	.14
Satisfaction with patient portal (excellent),^d^ n (%)	1654/3653 (45.28)	299/993 (30.1)	.001

^a^ In total household income from all sources before taxes.

^b^ Rating of overall health was captured as excellent (5), very good (4), good (3), fair (2), or poor (1).

^c^ The selected problems included hypertension, hyperlipidemia, diabetes, cancer (any), coronary artery disease, congestive heart failure, asthma, osteoarthritis, rheumatoid arthritis, and depression.

^d^ Satisfaction with patient portal was captured as excellent (5), very good (4), good (3), fair (2), or poor (1).

Of the 4109 patients who were aware of the AVS, 2248 (54.71%) reported that they accessed the AVS through the patient portal within 5 days of the visit and 1805 (43.93%) did not access the AVS (56/4109, 1.4% did not respond). The top two reasons provided for not accessing the AVS through the portal were did not have a need for the AVS (45.43%, 820/1805) and did not remember that AVS was available through the portal (31.63%, 571/1805). Another 14.68% (265/1805) reported that they had received a copy of the AVS from their doctor’s office. Only 3.49% (63/1805) reported that they did not know how to access the AVS through the patient portal.

In total, 61.30% (1378/2248) of patients who accessed the AVS through the patient portal were female and 61.61% (1112/1805) of patients who did not access the AVS through the portal were female (Table 3). Mean age of patients who accessed the AVS was 56.5 (SD 14.1) years and mean age was 56.3 (SD 13.8) years for patients who did not access the AVS. In all, 90.52% (2035/2248) of patients who accessed the AVS and 90.47% (1633/1805) of patients who did not access the AVS were white. Of patients who accessed the AVS, 66.08% (1325/2005) had a 4-year college degree or more compared to 69.22% (1102/1592) of patients who did not access the AVS. Also, 62.85% (1132/1801) of patients who accessed the AVS and 62.18% (878/1412) of patients who did not access the AVS had a total household income of US $75,000 or more. Patients who accessed the AVS had a greater proportion reporting a status as a married or unmarried couple (69.69%, 1391/1996) compared to patients who did not access the AVS (65.44%, 1032/1577, *P*=.007). Patients reporting health status of very good or excellent was similar in both groups (50.59%, 1022/202 in access group and 51.75%, 827/1598 in did not access group). Patients who accessed the AVS had a portal account for a greater number of years (mean 3.6, SD 3.3) compared to patients who did not access the AVS (mean 3.1, SD 3.1, *P*<.001). Patients who accessed the AVS also used the portal more than patients who did not access the AVS (mean 119, SD 221.5 sessions vs mean 79.1, SD 123.3 sessions, *P*<.001; mean 8.2, SD 20.8 messages sent via the portal vs mean of 6.6, SD 18.2 messages sent via the portal, *P*=.01). Patients who accessed the AVS reported greater satisfaction with the portal (excellent: 51.03%, 1032/2022) compared to patients who did not access the AVS (excellent: 38.00%, 602/1584, *P*<.001).

**Table 3:** Characteristics of respondents who accessed or did not access the after-visit summary (AVS).^a^

Characteristics	Accessed AVS n=2248	Did not access AVS n=1805	*P*
Gender (female), n (%)	1378 (61.30)	1112 (61.61)	.84
Age (years), mean (SD)	56.5 (14.1)	56.3 (13.8)	.73
Race (white), n (%)	2035 (90.52)	1633 (90.47)	.95
Education (≥4-year college degree), n (%)	1325/2005 (66.08)	1102/1592 (69.22)	.046
Income (≥US $75,000),^b^ n (%)	1132/1801 (62.85)	878/1412 (62.18)	.70
Marital status (married or unmarried couple), n (%)	1391/1996 (69.69)	1032/1577 (65.44)	.007
Health status (very good or excellent),^c^ n (%)	1022/2020 (50.59)	827/1598 (51.75)	.49
Selected problems on problem list,^d^ mean (SD)	2.2 (1.2)	2.1 (1.1)	.03
Years with patient portal account, mean (SD)	3.6 (3.3)	3.1 (3.1)	.001
Sessions with patient portal, mean (SD)	119.0 (221.5)	79.1 (123.3)	.001
Messages sent via patient portal, mean (SD)	8.2 (20.8)	6.6 (18.2)	.01
Satisfaction with patient portal (excellent),^e^ n (%)	1032/2022 (51.03)	602/1584 (38.00)	.001

^a^ Data presented for patients who were aware that the AVS was available through the patient portal.

^b^ In total household income from all sources before taxes.

^c^ Rating of overall health was captured as excellent (5), very good (4), good (3), fair (2), or poor (1).

^d^ The selected problems included hypertension, hyperlipidemia, diabetes, cancer (any), coronary artery disease, congestive heart failure, asthma, osteoarthritis, rheumatoid arthritis, and depression.

^e^ Satisfaction with patient portal was captured as excellent (5), very good (4), good (3), fair (2), or poor (1).

### Satisfaction With After-Visit Summary

We assessed patient satisfaction with the AVS by asking patients to rate the AVS on a scale from 1 (poor) to 5 (excellent). The mean satisfaction with the AVS was 3.9 (SD 1.12). Patients 65 years and older reported greater satisfaction with the AVS (mean 3.9, SD 1.1) than patients younger than 65 years of age (mean 3.8, SD 1.1, *P*=.02). Nonwhite patients reported greater satisfaction (mean 4.0, SD 1.0) than white patients did (mean 3.9, SD 1.1, *P*=.04). Patients who reported very good or excellent health status reported greater satisfaction with the AVS (mean 4.0, SD 1.1) than patients who reported other (poor/fair/good) health status (mean 3.8, SD 1.2, *P*=.02). Patients who had less than a 4-year college degree reported greater satisfaction (mean 4.1, SD 1.0) than patients who had a 4-year college degree or more (mean 3.8, SD 1.2, *P*<.001). We fitted a multiple regression (forced entry) with satisfaction with AVS as the dependent variable and sociodemographics and portal-related variables as predictors (Table 4). Satisfaction with the patient portal was the most significant predictor of satisfaction with the AVS (beta=.679, *P*<.001). The number of portal sessions was also a significant predictor of the AVS (beta=–.095, *P*<.001). Among sociodemographic variables, age, gender, race, and education were significant predictors of satisfaction with AVS. Finally, patient-reported health status was a significant predictor of satisfaction with AVS.

### The Theory of Planned Behavior

#### Components of Theory of Planned Behavior

Table 5 shows results of reliability analyses for the three main factors in TPB and for the outcome variable of behavioral intention. Cronbach alpha was very good for all the factors. We created scales for each of the factors using a mean of the scores of the items pertaining to each factor.

**Table 4:** Multiple regression with satisfaction with after-visit summary as the dependent variable.

Predictors	Beta (standardized coefficient)	*P*
(Constant)		<.001
Age	.039	.002
Gender	–.036	.003
Race	–.031	.008
Health status	.037	.003
Education	–.069	<.001
Income	–.018	.19
Marital status	–.017	.20
Years with patient portal account	–.003	.82
Sessions with patient portal	–.095	<.001
Messages sent via patient portal	.002	.89
Satisfaction with patient portal	.679	<.001

**Table 5:** Reliability analysis for attitude, perceived norm, perceived behavioral control, and behavioral intentions.

TPB factor	Items	Cronbach alpha
Attitude	My accessing the visit summary report via patient gateway within 5 days of the visit is bad/good, pleasant/unpleasant, harmful/beneficial, useless/useful	.87
Perceived norm	Most people who are important to me think that I should access the visit summary report via patient gateway within 5 days of the visit; most people whose opinions I value would approve of my accessing the visit summary report via patient gateway within 5 days of the visit; most people I respect and value will access the visit summary report via patient gateway within 5 days of the visit; most people like me will access the visit summary report via patient gateway within 5 days of the visit	.86
Perceived behavioral control	I am confident that I can access the visit summary report via patient gateway within 5 days of the visit; my accessing the visit summary report via patient gateway within 5 days of the visit is completely up to me; if I really wanted to, I can access the visit summary report via patient gateway within 5 days of the visit; I have complete control over whether or not I access the visit summary report via Patient Gateway within 5 days of the visit	.88
Behavioral intention	I intend to access the visit summary report via patient gateway within 5 days of the visit; I will access the visit summary report via patient gateway within 5 days of the visit; how likely or unlikely is it that you will access the visit summary report via patient gateway within 5 days of the visit; I plan to access the visit summary report via patient gateway within 5 days of the visit	.96

Table 6 shows results for behavioral belief strength, outcome evaluation, and the product of behavioral belief strength and outcome evaluation for the behavioral beliefs included in this study. Mean belief strength was on the positive side for all the behavioral beliefs. The most strongly held beliefs were patients’ ability to track visits and tests, and patients’ having medical information more readily accessible. The two beliefs that were lowest in strength were clarifying issues with their doctor and reinforcing doctor’s instructions. These two beliefs also had the lowest outcome evaluation and the greatest variation in belief strength and outcome evaluation. The mean behavioral belief strength×outcome evaluation products show that the three beliefs with the strongest positive impact on attitude were being able to track visits and tests, having medical information more readily accessible, and a more efficient way to obtain medical information. The correlations between behavioral belief strength×outcome evaluation and attitude were positive and significant and ranged from .45 to .52.

Table 7 shows results for normative belief strengths, motivation to comply, and the product of normative belief strengths and motivation to comply for the three referents included in the study. Mean normative belief strength was positive for all three referents. The strongest belief strength was associated with the patients’ doctor. Motivation to comply was also positive with the strongest motivation to comply associated with the patient’s doctor. Correlations between normative belief strength×motivation to comply and injunctive norm were all positive and significant.

Table 8 shows results for control beliefs, power of factor, and the product of control beliefs and power of factor. The strongest control belief was that the patient will have access to the Internet within 5 days of the visit. This was followed by the control belief “it will be easy for me to access the AVS via the patient portal” and the belief “I will remember the user ID and password for the patient portal.” Correlations between the product of control belief and power of factor and perceived behavioral control were all positive and significant. The strongest correlation was for the control belief “it will be easy for me to access the AVS via the patient portal.”

**Table 6:** Behavioral beliefs, outcome evaluation, and correlations with attitude.

My accessing the visit summary report of my office visit via patient gateway within 5 days of the visit will result in:	Belief strength, mean (SD)	Outcome evaluation, mean (SD)	Belief strength×outcome evaluation		
			Mean (SD)	*r* ^a^	*P*
My obtaining medical information (laboratory results, test results) in a more timely manner	2.38 (1.18)	2.55 (0.93)	6.62 (3.60)	.48	<.001
My having up to date medical information	2.40 (1.16)	2.57 (0.94)	6.74 (3.50)	.50	<.001
My having medical information more readily accessible	2.48 (1.07)	2.58 (0.92)	6.93 (3.36)	.50	<.001
Being able to clarify issues with my doctor	2.21 (1.27)	2.42 (1.10)	6.17 (3.73)	.45	<.001
My being able to track my visits and tests	2.53 (1.00)	2.60 (0.87)	7.06 (3.23)	.52	<.001
My being able to view all my medical information in one location	2.38 (1.21)	2.53 (1.04)	6.74 (3.58)	.45	<.001
Reinforcing my doctor’s instructions	2.18 (1.30)	2.37 (1.12)	6.11 (3.76)	.45	<.001
A more efficient way to obtain my medical information	2.41 (1.14)	2.54 (0.98)	6.84 (3.41)	.49	<.001

^a^ Correlation between behavioral belief strength×outcome evaluation and attitude.

**Table 7:** Injunctive normative beliefs, motivation to comply, and correlations with injunctive norm.

My _____ thinks that I should access the visit summary report of my office visit via patient gateway within 5 days of the visit	Normative belief strength, mean (SD)	Motivation to comply, mean (SD)	Normative belief strength×motivation to comply		
			Mean (SD)	*r* ^a^	*P*
Spouse/Partner	0.92 (1.77)	0.57 (1.77)	2.49 (3.92)	.31	<.001
Doctor	1.66 (1.55)	2.12 (1.24)	4.38 (4.27)	.45	<.001
Nurse	1.19 (1.59)	1.49 (1.46)	2.87 (3.97)	.38	<.001

^a^ Correlation between normative belief strength×motivation and injunctive norm.

**Table 8:** Control beliefs, power of factors, and correlations with perceived behavioral control.

Belief	Control belief strength, mean (SD)	Power of factor,^a^ mean (SD)	Control belief strength×power of factor		
			Mean (SD)	*r* ^b^	P
I will receive a reminder email after the office visit that the visit summary report is available via patient gateway	2.08 (1.42)	6.07 (1.42)	15.02 (7.94)	.21	<.001
I will have all my medical information in the visit summary report after the office visit	2.08 (1.33)	6.08 (1.33)	14.57 (8.08)	.27	<.001
I will have access to the Internet within 5 days of the office visit	2.76 (0.70)	6.76 (0.70)	17.85 (6.59)	.29	<.001
I will remember my patient gateway user ID and password	2.51 (0.98)	6.51 (0.98)	16.62 (7.28)	.27	<.001
I will understand the information in the visit summary report	2.39 (0.93)	6.39 (0.93)	14.07 (9.26)	.21	<.001
It will be easy for me to access the visit summary report via patient gateway	2.64 (0.81)	6.64 (0.81)	16.95 (6.90)	.33	<.001

^a^ Power of factor measured on a scale from 1 (likely) to 7 (unlikely) and then reverse coded.

^b^ Correlation between control belief × power of factor and perceived behavioral control.

### Predictors of Behavioral Intention

We employed hierarchical multiple regression to assess predictors of behavioral intention with respect to accessing the AVS through the patient portal and to test the TPB model (Table 9). Given the TPB model, we entered attitude, perceived norm, and perceived behavioral control in the first model. In the second and third models, we entered variables external to the TPB model: variables related to patient portal use were entered in the second model and sociodemographic variables and health status were entered in the third model. The first model consisting of the direct predictors of behavioral intention, attitude, perceived norm, and perceived behavioral control was significant and accounted for 56.7% of the variance in behavioral intention. The second model with portal variables was also significant but added only 5%. Years with portal account, number of sessions, and satisfaction with the AVS were significant predictors in this model. The third model was also significant and added another 3%. Age was the only sociodemographic variable that was significant in this model (beta=.049, *P*<.001*)*.

**Table 9:** Hierarchical multiple regression analysis predicting behavioral intention.

Factors		Beta (standardized coefficient)	*R* ^*2*^ change	*P*
**TPB factors**			.567	<.001
	(Constant)			<.001
	Attitude	.432		<.001
	Perceived norm	.291		<.001
	Perceived behavioral control	.206		<.001
**TPB factors and patient portal factors**			.005	<.001
	(Constant)			<.001
	Attitude	.415		<.001
	Perceived norm	.285		<.001
	Perceived behavioral control	.197		<.001
	Number of years with patient portal account	.040		.001
	Number of sessions with patient portal	.039		.003
	Number of messages sent via patient portal	–.008		.54
	Satisfaction with patient portal	.003		.87
	Satisfaction with AVS	.044		.006
**TPB factors, patient portal factors, sociodemographics, and self-reported health status**			.003	.004
	(Constant)			<.001
	Attitude	.412		<.001
	Perceived norm	.284		<.001
	Perceived behavioral control	.197		<.001
	Number of years with patient portal account	.030		.02
	Number of sessions with patient portal	.035		.008
	Number of messages sent via patient portal	–.004		.75
	Satisfaction with patient portal	.003		.85
	Satisfaction with AVS	.042		.009
	Age	.049		<.001
	Gender	.019		.09
	Race	.012		.28
	Health status	–.001		.91
	Education	–.011		.33
	Income	–.008		.53
	Marital status	.010		.40

## Discussion

In this study, we assessed patient awareness and access of the AVS provided through a patient portal. A large majority of users of the portal were aware that the AVS was available through the portal. Of those who were aware, just over half reported that they accessed the AVS through the portal within 5 days of an office visit. There were no differences between the groups with respect to sociodemographics (eg, age, gender, race, and self-reported health status). Education and income were related to awareness of the AVS but in a reverse direction than expected: users of the portal with more education and higher income were more likely to be unaware of the availability of the AVS through the portal. However, these differences did not carry over to the access of the AVS through the portal. Given these findings on race, income, and education, our study does not find a digital divide in the case of both awareness and access of the AVS through the patient portal. In a previous study on the digital divide associated with the adoption and use of the patient portal, we found that the digital divide did not carry over to portal use, specifically the relationship between income and the frequency of secure messaging through the portal [15]. In this study, accessing the AVS through the patient portal yields a similar finding: once patients have adopted the portal and are using it, issues of digital divide may not persist at least with respect to some of the functionality of the portal. However, there is a need for additional research on the digital divide with respect to use of portal functionality.

Although previous studies have reported high levels of satisfaction overall with patient portals, there has been little research on satisfaction with the use of specific portal functionality such as the AVS. Our study found a high level of satisfaction with the AVS similar to the finding by Pavlik and colleagues [10]. At the same time, overall satisfaction with the patient portal was positively and significantly related to both awareness and access of the AVS through the patient portal. Satisfaction with the portal was also the most important predictor of satisfaction with the AVS. We agree with the need for empirically measuring a quality indicator such as patient satisfaction with the portal because it appears to be a driver of patient satisfaction with specific functionality of the portal such as the AVS [4].

We do not know of other work that has previously applied TPB to assess patients’ beliefs and predict behavioral intention toward accessing their AVS through a patient portal and our findings support the use of the theoretical model in this area. The correlations between beliefs about accessing the AVS through the patient portal and the major determinants of the TPB model (attitude, perceived norm, and perceived behavioral control) were positive and significant, and similar to those reported in studies on other health behaviors. Ajzen [16] reported mean correlations between the expectancy-value index of beliefs and a direct attitude measure ranging from .50 to .53 based on the findings of two meta-analyses of studies applying TPB to health behaviors. The correlations in our study (Table 5) ranged from .45 to .50 with a mean of .48. McEachan and colleagues [14] conducted a meta-analysis of 237 prospective tests of the TPB applied to health behaviors. They reported a corrected (for sampling and measurement error) mean correlation of .57 between attitude and intention, a mean correlation of .54 between perceived behavioral control and intention, and a mean correlation of .40 between subjective norm and intention. In our study, correlation between attitude and intention was .65, perceived behavioral control and intention was .47, and perceived norm and intention was .58**.** The prediction of behavioral intention to access the AVS through the patient portal from attitude, perceived norm, and perceived behavioral control yielded similar findings to other applications of TPB in the health arena. Ajzen [16] reported a meta-analysis showing the mean multiple correlation between the three major determinants of TPB and behavioral intention to range from .59 and .66. In terms of prediction of intention, McEachan and colleagues [14] reported that 44.3% of the variance in intention was accounted by attitude, subjective norm, and perceived behavioral control. Attitude was the strongest predictor (beta=.35) followed by perceived behavioral control (beta=.34) and subjective norm (beta=.15). In our study, 56.7% of the variance in intention was accounted by attitude, perceived norm, and perceived behavioral control. Attitude was the strongest predictor of intention (beta=.43) followed by perceived norm (beta=.29) and perceived behavioral control (beta=.21).

In terms of specific beliefs, our study found that behavioral beliefs related to patient access of information through the AVS, specifically the ability to track visits and tests, have medical information more readily accessible, and obtain medical information more efficiently, were more important than beliefs about patient engagement in their health care, such as clarifying issues with their doctor or reinforcing instructions. This finding is similar to the finding obtained in studies on patients accessing their doctor’s notes that patients value access to their information [17-20]. In the VA Open Notes study, patients accessed their doctor’s notes to be better prepared for clinic visits, remember their care plan better, and feel more in control of their health [17,18]. Delbanco and colleagues [19,20] also found patients accessed their doctor’s notes to be better prepared for future visits and have a greater sense of control over their health. In their organization, Ralston and colleagues [9] noted the AVS may serve patient’s information and care needs better because it provides a focused plan of care combined with educational materials hyperlinked to other sources. The question of when patients prefer to access their AVS compared to their doctor’s notes through a patient portal, and the relative value of these uses of the portal, are important topics for future research.

In the case of normative and control beliefs, the strongest normative belief and motivation to comply were associated with the patient’s doctor. Patients believe that their doctor thinks they should access the AVS and they want to do what their doctor thinks. The importance of clinician encouragement of patients using online tools such as patient portals has been identified in several studies [9,11,21]. Our study finds that clinicians also have an important role in encouraging patients to access specific functionality of portals such as the AVS. In the case of control beliefs, the ease of accessing the AVS through the patient portal was identified as an important belief. The importance of ease of access and use of patient portals has been well documented in empirical and scoping studies [11,12,22,23]. Our study finds that even after patients have adopted patient portals, ease of access of specific functionality of the portals can facilitate or impede intention and behavior to use the functionality.

This study had two objectives: (1) assess the characteristics of patients who are aware of and access the AVS through a patient portal and (2) apply TPB to evaluate beliefs, attitude, perceived norm, perceived behavioral control, and predict behavioral intention of patients toward accessing the AVS through a patient portal. A majority of users of a patient portal selected for this study were aware that the AVS was accessible through the portal, but almost a third did not remember that the AVS was available and therefore did not access it. Patients may need to be reminded that the AVS is accessible through the portal at the time they leave their office visit, especially if patient preference is to receive information through the patient portal. In terms of patient characteristics, we did not find evidence of a digital divide with respect to income or education in either awareness or access of the AVS in our portal users. On the other hand, portal users in one setting who accessed their doctor’s notes were more educated than those who did not access the notes [18]. Additional research is needed on issues of digital divide with respect to different uses of the patient portal, such as accessing the AVS or doctor’s notes.

With respect to the meaningful use of EHR incentive program, two goals were envisioned related to EHR functionality such as the AVS [1,2]: (1) provide patients with timely and efficient access to their health information and (2) motivate patients to engage in their health care. Other studies have also identified patient engagement in their health care (eg, shared decision making with their doctor) as an important purpose of the AVS [24]. Our study found that patients had stronger beliefs about the AVS with respect to timely and efficient access of information than with engaging in their health care. This finding may reflect patients’ value of the AVS as a permanent personal record to review whenever the need arises [25]. On the other hand, it is possible that the use of the AVS to engage patients in their health care is not being promoted. Pavlik and colleagues [10] noted the need for concerted efforts to remind patients of important information available through the AVS than simply providing the AVS to patients. Such efforts can lead to patient activation and the use of information by patients to undertake recommended treatment plans and self-management, both of which are important goals for the AVS [24]. Although Stage 1 of the MU program included a core requirement for the provision of the AVS, Stage 2 of the MU program no longer includes this core requirement. This is unfortunate given the value of the AVS for providing timely and efficient access of information as reported by patients in our study.

We found TPB to be a suitable theoretical model to predict behavioral intention of patients toward accessing the AVS through a patient portal. Our findings match applications of TPB for predicting intention with respect to a variety of other health behaviors. Thus, this study provides an important contribution to the application of theoretical models to the study of patient portals and extends some of the prior theoretical work on this topic [11,12]. Beyond its theoretical contribution, the application of TPB can suggest interventions that are relevant to practitioners. For example, Fishbein and Ajzen [13] recommend interventions that target and change relevant salient beliefs or make new beliefs salient in support of recommended behavior. Our study found that the strongest behavioral beliefs related to accessing the AVS through the patient portal are those related to tracking visits and tests, and having medical information more readily and efficiently accessible. Our study also found that doctors are an important social agent for patients with respect to accessing the AVS. For those patients who are not accessing the AVS through the patient portal, a simple intervention that organizations can implement would be to encourage doctors and support staff to discuss with patients the advantages of accessing the AVS through the portal, such as tracking visits and tests. This intervention would also help those patients who do not remember that the AVS is available through the portal. Similarly, doctors can ask patients to use the AVS for clarifying instructions and engaging in shared decision making. Engaging patients in a dialog about the use of the AVS may also help facilitate two important factors identified by us in a survey of physician beliefs about the AVS: (1) enhancing physician satisfaction with the AVS and (2) promoting positive beliefs about the effect of the AVS on patient outcomes and the care the physician personally delivers [26].

Although this study yielded valuable insights into awareness and access of the AVS through a patient portal and the application of TPB to this area, it is associated with some limitations. The response rate in our study was low. We relied on a self-report of patients accessing the AVS through the patient portal. With respect to the time component, we chose 5 days from the office visit as the period within which the patient accessed the AVS. There is a need to assess other time periods after an office visit in which the patient could access the AVS. The study was conducted in the setting of a Northeast academic medical center and the results may not be generalizable to other regions and patient populations. However, we have no reason to suspect that the beliefs identified in this study and their respective strengths would differ across different institutional settings in which the AVS is available through a patient portal (academic medical center vs other) or across different platforms (homegrown vs vendor patient portals).

## Appendix

Multimedia Appendix 1 Survey Items.

## Abbreviations

AVS: after-visit summary
EHR: electronic health record
HITECH: Health Information Technology for Economic and Clinical Health
MU: Meaningful Use
TPB: Theory of Planned Behavior
