# Supplementary material for: Awareness and Use of the After-Visit Summary Through a Patient Portal: Evaluation of Patient Characteristics and an Application of the Theory of Planned Behavior
Source: J Med Internet Res. 2016 Apr 13;18(4):e77. doi: 10.2196/jmir.5207 (PMC4848387; doi:10.2196/jmir.5207)
Supplement: Multimedia Appendix 1 [file jmir_v18i4e77_app1.pdf]

Appendix 1

Survey items

A. **TPB Items**

Behavioral beliefs and attitudes

In our survey we captured behavioral beliefs with accessing the AVS through the patient portal within five days of the visit as consequences associated with the access such as: obtaining medical information (lab results, test results) in a more timely manner; having up to date medical information; being able to view all of the medical information in one location; and reinforcing the doctor's instructions. Additionally, in TPB, behavioral beliefs are captured through two scales: a measure of the belief strength, and a measure of the evaluative aspect of the belief or an outcome evaluation. For each item, these two scales are multiplied to create a behavioral belief score for that item. The following is an example of a behavioral belief item, reinforcing doctor's instructions, and the belief strength and outcome evaluation of associated with this item on our survey:

Belief strength[a]: My accessing the visit summary report of my office visit via Patient Gateway within five days of the visit will result in reinforcing my doctor's instructions.

Strongly O O O O O O O Strongly
Agree +3 +2 +1 0 -1 -2 -3 Disagree

Outcome evaluation:
Reinforcing my doctor's instructions is:

Good O O O O O O O Bad
+3 +2 +1 0 -1 -2 -3

(a At our organization, the AVS was called the visit summary report when this study was conducted and we chose to use that term in our survey items as patients were familiar with it. Patient Gateway refers to the name of our patient portal).

We captured attitudes as overall evaluations of accessing the AVS through the portal using bipolar evaluative dimensions: bad/good, pleasant/unpleasant, harmful/beneficial, and useless/useful. The following is an example of an evaluative dimension in our survey:

My accessing the visit summary report via Patient Gateway within five days of the visit is:

Bad ○ ○ ○ ○ ○ ○ ○ Good
-3 -2 -1 0 +1 +2 +3

In TPB, attitude follows directly and automatically from beliefs through the expectancy-value model:

$A \ \alpha \ \sum b_i e_i$

A = attitude toward accessing the AVS through the portal

b_i = strength of the belief that accessing the AVS through the portal has attribute i (e.g., reinforcing doctor's instructions)

e_i = evaluation of attribute i

Normative beliefs and perceived norm

In this study, we captured normative beliefs as injunctive normative beliefs (beliefs about what others think the patient should do). In terms of injunctive normative beliefs, we focused on specific social agents such as the patient's doctor rather than a generalized social agent. The three social agents we selected for this study were: spouse or partner, doctor, and nurse. Additionally, we captured patient's motivation to comply with the social agent as the patient may not necessarily follow what the social agent recommends. The following is an example of an item on the survey with the doctor as the social agent.

<u>Belief</u>: My doctor thinks that I should access the visit summary report of my office visit via Patient Gateway within five days of the visit

Strongly ○ ○ ○ ○ ○ ○ ○ Strongly
Agree +3 +2 +1 0 -1 -2 -3 Disagree

Motivation:

When it comes to accessing the visit summary report of my office visit via Patient Gateway within five days of the visit, I want to do what my doctor thinks I should do

Strongly Agree	O	O	O	O	O	O	O	Strongly Disagree
	+3	+2	+1	0	-1	-2	-3	

Perceived norms were captured as overall evaluations with respect to attributes such as important to the patient or like the patient. The following is an example of a perceived norm item on the survey:

Most people like me will access the visit summary report via Patient Gateway within five days of the visit

Strongly Agree	O	O	O	O	O	O	O	Strongly Disagree
	+3	+2	+1	0	-1	-2	-3	

Perceived norm follows from injunctive normative beliefs through the following model:

$Ni \; \alpha \; \sum n_i m_i$

N_i = Perceived norm

n_i = injunctive normative belief about referent i

m_i = motivation to comply with referent i

Control beliefs and perceived behavioral control

Control beliefs were captured through factors that facilitate or inhibit the behavior under study. These factors are also associated with power of control.

Belief: Remembering my Patient Gateway userid and password would enable me to access the visit summary report via Patient Gateway within five days of the visit

Strongly Agree	O	O	O	O	O	O	O	Strongly Disagree
	+3	+2	+1	0	-1	-2	-3	

Power of control

I will have access to the Internet within five days of the office visit

Likely ○ ○ ○ ○ ○ ○ ○ Unlikely
1 2 3 4 5 6 7

Perceived behavioral control was assessed through overall evaluations of the patient's control in accessing the AVS through the patient portal. The following is an example of an item on our survey.

I am confident that I can access the visit summary report via Patient Gateway within five days of the visit

Strongly ○ ○ ○ ○ ○ ○ ○ Strongly
Agree +3 +2 +1 0 -1 -2 -3 Disagree

Behavioral intention

Our dependent variable in this study was behavioral intention to access the AVS through the patient portal within five days of the visit. We captured behavioral intention using items measured on Likert scales such as "I intend to access the after visit summary" and "I plan to access the after visit summary".

B. **Other items**

For technical reasons we were unable to gather data from the patient portal system on whether a patient accessed an AVS associated with an office visit. As a result we had to rely on patients' self-report that they accessed the AVS associated with an office visit. Our survey instrument included two questions pertaining to this: (1) Are you aware that you can access your visit summary report from your doctor's visit via Patient Gateway (Yes/No)?
and (2) Did you access your visit summary report via Patient Gateway within five days of the office visit (Yes/No)?
We also gathered the following socio-demographic data through our survey: education, income, and marital status. We asked patients for a self-report of their health status. Finally, we asked patients to rate their satisfaction with the patient portal, and their satisfaction with the AVS.

In the case of gender, age, and race, we relied on data from the Partners scheduling and billing systems. We also gathered data from the Partners patient portal system on patient experience with the portal: number of years with a portal account, number of sessions with the portal, and number of messages sent via the portal.
